# Supplementary material for: Are carbon emissions trading and green financial instruments synergistic? -Comprehensive quantitative research based on content analysis
Source: PLoS One. 2024 Mar 7;19(3):e0298601. doi: 10.1371/journal.pone.0298601 (PMC10919601; doi:10.1371/journal.pone.0298601)
Supplement: S1 Data — (ZIP) [file pone.0298601.s001.zip › Data/地级市Z_F工作报告.zip!/╣π╢1⁄2/╔ε█┌╩╨/└┤╫╘╣1⁄2╓┌║┼í╛┬φ┐╦╩2╛▌═°í┐_2013.docx]

各位代表：

　　现在，我代表深圳市人民政府向大会作政府工作报告，请予审议，并请各位政协委员和其他列席人员提出意见。

　　一、2012年工作回顾

　　2012年，是深圳科学发展取得显著成效的一年，是深化改革、扩大开放取得重要进展的一年，是全市人民团结一致、攻坚克难、奋力前行的一年。面对复杂严峻的外部环境和转型升级的内在压力，在党中央国务院、省委省政府和市委的坚强领导下，我们围绕主题主线，以“深圳质量”为标杆，立足当前，着眼长远，采取一系列前瞻性、针对性举措，全力以赴强化创新驱动、加快转型升级、推进社会建设、努力改善民生，完成了市五届人大三次会议确定的主要目标任务，实现了有质量的稳定增长、可持续的全面发展。

　　全年本市生产总值1.295万亿元，增长10%；固定资产投资2314.43亿元，增长12.3%；进出口总额4667.85亿美元，增长12.7%，跃居内地大中城市首位，其中出口2713.7亿美元，实现二十连冠；全口径公共财政收入4502亿元，其中地方公共财政预算收入1482亿元，增长10.6%；居民消费价格涨幅为2.8%，低于预期目标1.2个百分点。

　　（一）质量提升引领经济发展新跨越。主要指标实现“三个突破”，本市生产总值突破2000亿美元，社会消费品零售总额突破4000亿元，人均可支配收入突破4万元。经济效益持续提高。人均GDP接近2万美元，每平方公里产出超过1亿美元，税收占财政收入比重达90%，均居内地大中城市前列。战略性新兴产业和现代服务业成为双引擎。重点扶持战略性新兴产业项目2000多个，六大战略性新兴产业总体增速为经济增速两倍以上，占本市生产总值比重超过25%，我市成为国内战略性新兴产业规模最大、集聚性最强的城市之一。服务业占本市生产总值比重提高到近56%，现代服务业占服务业比重达68%。自主创新成为经济增长内生动力。全社会研发投入占GDP比重提高到3.81%，居全国领先水平，PCT国际专利申请量8024件，占全国的40.3%，连续9年居全国首位。高新技术产品产值1.29万亿元，其中具有自主知识产权的达61%。内需拉动作用明显增强。消费、投资增速分别高于GDP增速6.5和2.3个百分点。在经济总量取得突破的同时，资源能源消耗首次出现“三个总量下降”。用水总量、汽柴油销售量、制造业用电量分别下降0.61%、1.79%和1.06%。这标志着我市转变经济发展方式取得重要成效。

　　（二）结构优化增强经济发展后劲。及时出台支持扩大出口和内销的稳定经贸发展33条、金融支持实体经济24条、促进产业转型升级36条、鼓励总部企业集聚发展29条等政策措施，组织开展政策宣讲40多场，建立重大项目分级协调和领导挂点服务企业机制，全年共协调重大项目284个，挂点服务重点企业1480家，帮助企业解决遇到的困难和问题，实现经济增长逐季加速，产业结构不断优化。制造业高端化步伐加快。支持产业转型升级项目693个，淘汰“两高一低”企业1575家。先进制造业占规模以上工业增加值的比重近70%，服装、钟表、黄金珠宝等产业的品牌优势进一步显现。我市累计拥有“中国驰名商标”103件，成为国内首个突破百件的副省级城市。现代服务业快速发展。金融业总资产突破5万亿元，深交所全年新增上市企业129家，继续位居全球第一；港口集装箱吞吐量近2300万标箱，连续10年居全球第四。投资结构改善。社会投资占总投资比重提高到78%，原特区外投资占总投资比重提高到72%。外贸结构优化。服务贸易出口增长37.6%，是出口整体增速的3.5倍，进料加工出口增速高于来料加工32个百分点，超过2000家来料加工企业实现不停产转型。总部经济加快集聚。华润集团中国内地总部、海信集团南方总部、中国电子国际总部等18家总部落户深圳，平安、华为、招商银行等进入世界500强，在深投资的世界500强企业累计达189家。重大产业项目加快推进。华星光电提前达产，旭硝子、深超光电等项目和全球首条超材料生产线投产，长安标致雪铁龙汽车项目进入设备安装调试阶段。

　　（三）自主创新提升核心竞争力。国家创新型城市建设取得重大进展。大亚湾中微子实验室发现中微子“第三种振荡”，入选美国《科学》杂志年度全球十大科学突破，华大基因科学家王俊入选英国《自然》杂志年度全球科学界十大人物。深圳成为获得这两项殊荣的唯一中国城市，我们倍感光荣和自豪！创新载体建设加速。国家超级计算深圳中心投入运行，国家基因库动工建设，新增国家、省、市级重点实验室、工程实验室、工程中心、技术中心等180家，累计757家。科技创新向前沿和高端迈进。第四代移动通信、基因测序、超材料、新能源汽车等技术处于世界前沿，光启研究院专利申请量占全球超材料领域的85%以上，华为、中兴在LTE技术领域的基本专利占全球的1/5。深圳承担的国家重大科技专项、国家自然科学基金立项数均增长35%。我市企业主导或参与制定国际、国家和行业标准416项，其中国际标准137项。创新生态体系更趋完善。出台建设国家自主创新示范区“1＋10”文件，强化创新政策支撑。新型研发机构加快推进知识创新、技术创新和产业化。北斗卫星导航、移动互联、超材料等产学研资联盟积极开展协同创新，战略性新兴产业集群不断崛起。金融服务科技创新的能力进一步增强，新增股权投资企业1450家，累计3500家以上。继续实施“孔雀计划”，引进高层次海外人才团队16个。成功举办第14届高交会和第11届国际人才交流大会。

　　（四）绿色低碳推动城市可持续发展。预计万元GDP能耗降幅超过4.25%，万元GDP水耗下降11.7%，化学需氧量、氨氮、二氧化硫、氮氧化物等年度减排任务超额完成。节能减排成效显著。新建绿色建筑292万平方米，累计达1265万平方米，居全国前列。全年新增建筑节能48万吨标准煤。全年推广新能源汽车2100辆，累计超过5000辆，继续位居全球城市首位。国际低碳城列入中欧可持续城镇化合作旗舰项目，启动区建设稳步推进。落实深圳经济特区碳排放相关法规，开展碳排放核查，碳排放交易准备工作进展顺利。资源循环利用水平提高。新建污水管网323公里，城市污水日处理能力达到469.5万吨，新增79.5万吨。日污水再生利用量达到136万吨，增长13.3%。建筑废弃物再利用总量350万吨，比上年提高16.6%。垃圾焚烧发电量4.5亿度，居全国第一。启动500个小区垃圾减量分类试点。生态宜居城市建设加快。大力开展城市绿化提升行动，新建和整理绿道1012公里，建设142公里、4.4万亩生态景观林带。新建改建公园64个，公园总数达841个。集中整治机动车、电厂等PM2.5重点排放源，空气质量持续好转。全力推进跨界河流污染治理，龙岗河、坪山河、观澜河水质通过省达标考核。加强市容环境综合整治，通过第三次国家环保模范城复查。

　　（五）特区一体化加快城市功能提升。特区一体化建设三年计划实施以来，原特区外固定资产投资达4429亿元，完成计划的1.6倍，500米公交站点覆盖率从78%提高到90%，千人总体学位数从78个提高到98个。光明、坪山、龙华加快从产业功能区向新城区转型，大鹏新区全面启动国际滨海生态旅游度假区规划建设，宝安、龙岗城市现代化水平进一步提高。基础设施更加完善。深圳机场T3航站楼主体工程完工，深圳北站、深圳东站等交通枢纽投入运营，轨道交通三期正式开工，深汕二期、布龙路二期等14条主干道建成通车，打通32条断头路。加快推进公交都市建设，新增、优化公交线路165条，新增公交专用道100公里、新一代公交候车亭855座、自行车道101公里，日均公交客流量突破千万人次。西气东输二线向深圳供气，新建市政燃气管道140公里，新增管道天然气居民用户10万户，500千伏鲲鹏变电站扩建等电网工程投入使用，清林径引水、公明供水调蓄工程进展顺利。大运中心、布吉污水处理厂等11项工程分获6项“鲁班奖”和5项国家优质工程奖。城市运行更加智能化。在全国率先建成第四代移动通信规模试验网，城市网络电视台影响力扩大，全市互联网、家庭宽带普及率分别达83%和80.8%，无线宽带覆盖率达85%。智能交通、数字城管、数字环保、科技强警等提高了城市智能化水平。土地空间利用更加集约化。积极推进城市更新和土地整备，开工城市更新项目60个，累计完成土地整备67平方公里，土地二次开发成为建设用地主要来源，一批旧工业区、旧商业区、城中村加快向新兴产业集聚地和现代化新社区转型。

　　（六）加强社会建设促进民生改善。市本级财政对重点民生领域投入达518亿元，增长14%。国家确定的“十二五”期间免费义务教育、基本药物制度、就业援助等涉及我市的65项地方基本公共服务全部落实。收入和社会保障水平持续提升。最低工资标准提高至每月1500元，最低生活保障标准提高至每人每月560元，人均可支配收入增速与经济增长同步，中低收入家庭收入增长快于高收入家庭。新增就业人数9.94万人，城镇居民登记失业率2.42%。出台住房保障制度改革创新纲要，新开工保障性住房3.8万套，竣工1.8万套，市区两级财政启动惠及20万人才的租房补贴，住房公积金归集人数位居全国第三。实行婚检及孕前优生健康检查和基本殡葬服务免费。各项社会事业快速发展。启动普惠性幼儿园试点70所，新增中小学和幼儿园学位4.5万个，享受免费义务教育学生61.8万名，国家级示范性高中比重达44.8%，建成国家级职业教育实训基地8个。南方科技大学正式建立，香港中文大学（深圳）获批筹建，中国资本市场学院挂牌成立，生物、新材料、先进技术等特色学院启动建设。香港大学深圳医院等一批医疗卫生设施投入使用，新建社会医院5家，新增病床3100张，新增省医学重点专科12个。试行先诊疗后结算、网络健康服务等方式，方便市民就医。社康中心服务能力增强，基层医疗机构诊疗量占全市67%。歌曲《走向复兴》等4部作品荣获全国“五个一工程奖”。深圳当代艺术馆和城市规划展览馆开工建设。公共文化体育服务体系日益完善，群众性文体活动广泛开展，全市172家公共图书馆与200台自助图书馆实现一证通。文博会规模和影响扩大，首届中国设计大展成功举办。第八届市运会暨首届体育大会顺利召开。深圳运动员在伦敦奥运会获2金3银，创历史最佳成绩。社会建设和管理取得新进步。实施社会建设“织网工程”和“风景林工程”，推进“一库两网”建设，推广社区公益服务等典型经验，建成社区服务中心214家，建立社区居民议事会744个，开通社区家园网48个。创新社会工作机制，提高社会工作服务水平，专业社工队伍超过4000人。“三打两建”工作成效显著，严厉查处商业贿赂，打掉一批欺行霸市团伙，查办制假售假案件2万余宗，社会信用体系、市场监管体系建设试点工作有序推进。加强社会治安立体防控，完善应急管理体系，社会治安、安全生产形势持续向好。加强自然灾害、事故灾难、公共卫生和社会安全等突发事件的预防和应对，启动城市公共安全评估及白皮书制定工作。健全食品药品安全监管体制机制。率先组建食品安全监管局，实行生产、流通、消费全过程监管，加大对非法养殖、私宰生猪和地沟油的打击力度。深入开展药品安全进社区活动，药品评价性抽样合格率达98.5%。社会文明呈现新风貌。“空谈误国、实干兴邦”等深圳“十大观念”深入人心，绿色出行、机动车礼让行人、排队、让座等文明行为蔚然成风，志愿者之城、爱心之城建设扎实推进，注册志愿者达46万人，扶贫济困、无偿献血、公益慈善广泛开展，成功举办首届中国慈展会，再次荣获全国文明城市称号。认真做好国防动员和双拥创建工作，连续五次获全国双拥模范城称号。老年人、妇女儿童、残疾人事业取得新进步。

　　（七）改革开放激发经济社会发展活力。坚持社会主义市场经济改革方向，着力构建政府、市场、社会三者相互协调、良性互动的格局。重点领域和关键环节改革迈出新步伐。商事登记制度改革取得重大突破，形成商事主体资格与经营资格相分离、审批与监管相统一、注册资本实行认缴制的商事登记管理新体系。投融资体制改革取得新成效，市属国资国企成为投融资改革的重要力量，社会投资快速增长。深化财政改革，优化财政支出结构，完善公共财政体系，营业税改征增值税试点工作顺利推进。开展土地管理制度改革综合试点，创新土地二次开发权益共享机制，完善城市更新的具体措施，出台优化空间资源配置促进转型升级的政策。公立医院管办分离、医药分开改革取得重大进展，在全国率先全面取消公立医院药品加成，门诊、住院药品费用平均下降9.3%和13.8%。加快户籍制度改革，建立非户籍人口入户的常态机制，实现高技能人才无障碍入户，有序推进基本公共服务均等化。社会组织管理改革进一步深化，直接登记范围扩大，各类社会组织超过5600家。公务员分类管理和聘任制改革、事业单位人事制度综合配套改革深入推进。连续四次荣获全国文化体制改革工作先进地区称号。对外开放和区域合作达到新水平。实际外商直接投资额52.3亿美元，现代服务业成为利用外资的主体。深圳境外投资新增22.1亿美元，增长155.5%，对外承包工程合同额185.4亿美元，增长42.5%。新增海法、亚特兰大等国际友好城市、友好交流城市9个，友城总数达44个。前海深港现代服务业合作区开发开放取得突破性进展，22条先行先试政策获国务院批复，前海咨询委员会正式成立，302家企业和机构被批准入区，注册资本达372.7亿元，跨境人民币贷款业务获批，建立粤港（前海）国际仲裁合作平台。深圳通与香港八达通实现互联互通。珠三角规划纲要年度考核32项全部达标。深莞惠一体化进程加快，基本实现通信同城化。深汕特别合作区建设全面启动。扶贫“双到”三年计划顺利完成，“双转移”工作被评为全省优秀，对口支援新疆、西藏和贵州等取得积极进展，经济特区服务全国的作用增强。

　　（八）提质提效强化政府服务。认真执行法律法规和人大各项决议，建立向人大报告和向政协通报工作的多层次常态化机制。办理人大代表建议712件、政协提案559件、市民建议7949件。加强政府自身建设，进一步转变政府职能，全面推行绩效管理，服务型政府建设取得新成效。继续深化行政审批制度改革，取消、调整市级行政审批事项113项，清理幅度达32.2%，行政审批事项网上办理率达97.1%。积极推进政府投资项目审批制度改革，实现跨部门协同办理和并联审批。“深圳政府在线”连续三年荣获全国政府网站绩效评估副省级城市第一名。加强重点领域立法工作，向人大提交立法议案8件，建议人大新制定法规4项、修改15项，制定政府规章8项，评估梳理规章150项。“法治政府建设指标体系”获中国法治政府奖。统计、计生、档案、司法、民族、宗教、口岸、外事、港澳、对台、侨务、民防、防震减灾、气象等工作质量进一步提高。

　　各位代表，过去一年，面对外部复杂环境的严峻考验，我们直面转型升级的挑战，打破传统发展路径的依赖，不为短期利益所惑，着眼长远，心无旁骛，主动作为，加强对经济形势的分析研判，密集出台政策，创新服务手段，加大协调力度，强化督察督办，在克难中前行、创新中发展、转型中升级，交出了一份来之不易的答卷。实践证明，“深圳质量”的理念完全符合党的十八大精神和科学发展观，有质量的稳定增长、可持续的全面发展之路完全符合深圳的客观实际，创新驱动、绿色低碳的发展战略完全符合城市当前和长远发展的需要。一年来的实践，更加坚定了我们以新的发展方式应对各种挑战，以“深圳质量”增创发展新优势的信心和决心，只要我们朝着这个方向坚定不移地走下去，深圳经济特区的道路一定会越走越宽广，深圳的未来一定会越来越美好！

　　过去一年的成绩，是在党中央国务院、省委省政府和市委坚强领导下，全市人民共同奋斗的结果。人民群众永远是我们事业的力量源泉，是深圳奇迹的创造者。在这里，我代表深圳市人民政府，向全市人民，向工作在不同岗位的广大干部群众，向给予我们不懈支持的人大代表、政协委员、各民主党派、工商联和社会各界人士，向中央和省各有关部门、中央和各省区市驻深单位，向驻深解放军、武警官兵，向所有关心支持深圳发展的港澳同胞、台湾同胞、海外侨胞、国际友人和在深工作的外国专家表示崇高的敬意和衷心的感谢！

　　回顾一年的工作，我们更加清醒地认识到，前进道路上还存在不少困难和挑战。一是资源能源和市场对外依存度高，易受外部经济环境影响，在经济发展迈上新台阶后，统筹推进转型升级、提升质量和稳定增长的难度加大。二是科技竞争日趋激烈，突破核心技术、提升源头创新能力、加强创新人才队伍建设的要求更加迫切。三是环境容量约束趋紧，推动绿色发展、循环发展、低碳发展的任务十分艰巨。四是在经济社会发展转型和深度城市化进程中，利益主体和诉求多元化，改革进入攻坚期、深水区，保障和改善民生，提高社会管理水平，促进和谐发展的要求更高。五是适应人民群众新期待，转变政府职能，改进工作作风，加强廉政建设还需付出更大努力。在今后工作中，我们将以对人民高度负责的态度，采取切实有效措施，克服困难，应对挑战，做出更好成绩。

　　二、2013年工作思路和重点

　　2013年，是深圳全面贯彻落实党的十八大精神的开局年，是实施“十二五”规划承前启后的关键年。习近平总书记视察广东深圳时，要求我们改革不停顿、开放不止步，努力成为发展中国特色社会主义的排头兵、深化改革开放的先行地、探索科学发展的实验区，率先全面建成小康社会、率先基本实现社会主义现代化。“三个定位、两个率先”为深圳经济特区新一轮发展明确了目标，指明了方向。

　　当前，外部形势错综复杂，全球经济进入深度转型调整期，低速增长的态势仍将延续，主要经济体需求疲软，各种形式的保护主义明显抬头，美日量化宽松货币政策和欧债危机深化的影响不可轻视。国内面临着增长速度放缓、结构调整难度加大和转型升级更加迫切等问题，但一系列宏观经济政策的调控作用显现，经济持续健康发展的积极因素增多。我们既要有强烈的忧患意识，更要有敏锐的机遇意识，善于在全球大背景下、全国大发展中谋划深圳的未来，发现机遇、抢抓机遇、用好机遇，脚踏实地，埋头苦干，在深化改革开放中实现新发展。

　　今年政府工作总体思路是：全面贯彻落实党的十八大精神和习近平总书记“三个定位、两个率先”的要求，高举中国特色社会主义伟大旗帜，紧紧围绕主题主线，牢固树立“深圳质量”理念，始终坚持有质量的稳定增长、可持续的全面发展，增强创新驱动发展新动力，实现改革开放新突破，构建现代产业新体系，打造绿色低碳新优势，开创社会建设新局面，提升文化发展软实力，强化全国经济中心城市辐射带动引领作用，加快现代化国际化先进城市建设，率先全面建成小康社会。

　　“深圳质量”是我市贯彻落实科学发展观的具体实践。有质量的稳定增长、可持续的全面发展是“深圳质量”的本质要求，必须贯彻到经济社会发展的各领域和全过程。

　　有质量，就是坚持质量第一，以质取胜，把有质量作为经济社会发展的根本取向，以质量引领发展，以质量提升核心竞争力，以更少的资源消耗、更低的环境成本，创造更多更好的发展成果，形成创新能力强、经济效益好、城市功能优、文明程度高的发展态势，打造质量型增长、内涵式发展的新格局。

　　稳定增长，就是尊重经济发展的客观规律，准确把握深圳经济发展的阶段性特征，深刻认识我市经济已由高速增长期进入平稳增长期，以科学合理的发展速度，为加快转型升级留有空间，为增强创新能力创造条件，为应对复杂多变的外部环境赢得主动，实现增长方式由要素驱动向创新驱动转变，实现更长时期更高质量的稳定增长。

　　可持续，就是坚持科学发展观的基本要求，牢固树立以人为本、尊重自然的理念，促进人与自然相和谐，经济社会发展与人口资源环境相协调；牢固树立科学求实的作风，既立足当前，更着眼长远，率先确立可持续的体制机制和政策导向，以发展方式的根本转变，实现城市的永续发展。

　　全面发展，就是坚持“五位一体”的总体布局，统筹协调经济、政治、文化、社会和生态文明建设，努力实现经济更发达、城市更美丽、文化更繁荣、社会更和谐、生活更幸福，促进人的全面发展与社会的全面进步。

　　今年全市经济社会发展主要目标是：本市生产总值增长9%；万元GDP能耗和水耗分别下降4.25%和5%；居民人均可支配收入与经济保持同步增长，城镇居民登记失业率控制在3%以内，居民消费价格涨幅控制在3.5%左右。重点做好以下八个方面工作：

　　（一）着力加快转型升级，实现更高质量的持续健康发展。转型升级是事关深圳前途命运的一场硬仗，当前的产业转型就是未来的有质量增长，无论经济形势多复杂，困难和压力有多大，转型升级的方向不能变，步伐不能停，力度不能减。一是以培育优质增量引领转型升级。加快发展战略性新兴产业，争取列入国家战略性新兴产业区域集聚发展试点，加快23个产业基地和集聚区建设，安排战略性新兴产业专项资金30亿元，扶持项目1000个，发展一批龙头企业，力争全年实现战略性新兴产业规模超过1.5万亿元，增加值占GDP比重30%左右。大力发展现代服务业，强化金融中心功能，打造财富管理中心和股权投资基金集聚地。加快建设平安保险、太平保险、招商证券等18座金融总部大厦，新引进金融机构15家以上。继续支持顺丰、朗华等物流企业做大做强，推动物流产业集约化、高端化、网络化和国际化。大力发展工业设计、检验检测等生产性服务业。推进国家服务外包示范市、电子商务示范城市和中国软件名城建设。进一步优化中小微企业发展环境，支持专、精、特、新的创新型中小微企业发展壮大。启动未来产业培育计划，在海洋经济、航空航天、生命健康等领域抢抓新的机遇。二是以优化存量推动转型升级。安排20亿元专项资金推动转型升级。根据各区域产业结构和发展阶段的差异性，因区施策。加快创新型产业用房建设、旧工业区改造，实施特色产业园培育工程，提高产业集聚化发展水平。加大技术和品牌创新力度，大力发展都市时尚型产业，推动服装、钟表、黄金珠宝等产业高端化，打造更多国内外知名品牌。促进信息化与工业化深度融合，提升装备制造、汽车制造、电子信息等产业国际竞争力。三是以高质量投资带动转型升级。落实国家促进民间投资“新36条”，促进投资稳定增长和结构优化，强化重大项目和上市企业用地保障，加快建设华为控股全球总部和百度、阿里巴巴国际总部、长安标致雪铁龙汽车等重大产业项目，推动坝光片区国际生物产业园、大沙河创新走廊、梅林电子商务产业带、华大基因生物产业基地建设。四是以扩大内需促进转型升级。抓住消费升级机遇，扩大深圳品牌影响力，推动商业模式创新，发挥“深商e天下”电子商务平台作用，积极发展服务消费、虚拟消费等新兴业态。优化本地消费环境，推进罗湖国际消费中心、福田中央商务区等核心区域建设，大力培育推广“深圳购物”、“深圳美食”。抓住新型城镇化深入推进的机遇，搭建平台，整合资源，加快技术、产品和服务外溢，扩大经济发展腹地。

　　（二）着力增强创新驱动发展新动力，加快建设国家自主创新示范区。创新是加快转变经济发展方式的核心推动力，是实现质量型增长的战略支撑。要以全球视野谋划和推动创新，加快建设创新环境优、创新能力强、创新效益好的国际创新中心。一是不断夯实创新基础。进一步加大科技投入，全社会研发投入占GDP比重力争达到3.9%。加快深圳国家基因库等重大科技基础设施建设，充分发挥国家超级计算深圳中心的作用。推进公共技术平台建设，吸引更多科研机构落户深圳，新增各级创新载体100家以上。加快培育新型研究机构，促进核心技术研发能力和科技成果产业化能力双提升。二是积极抢占科技创新制高点。力争在新一代信息技术、基因工程、超材料等优势领域，取得核心技术新突破。加强产业共性技术和关键技术的研发与应用，支持骨干企业、高等院校、科研机构联合组建研发平台，更多地承担和参与国家重大科技专项、重点技术攻关。全年组织实施50项以上高技术重大攻关项目。三是全方位优化创新生态。在关键领域组建一批产学研资联盟，推动协同创新。强化国际科技交流合作，推动开放式创新。促进科技与金融紧密结合，推动设立战略性新兴产业创投基金，争取新三板试点。加快国家知识产权示范城市建设，加强知识产权创造、运用、管理和保护，构建专业化、网络化的专利服务和标准服务体系。创新科技管理体制，完善科技创新评价体系。办好第十五届高交会。四是全力构筑创新人才高地。完善人才发展机制，高水平培养和高质量引进并举，集聚一批具有世界水准的科技领军人才、高技能人才、复合型人才和创新团队。加大招才引智力度，深入实施“孔雀计划”，引进海外高层次人才团队10个以上。进一步优化人才服务体系，全面实施人才安居工程。

　　（三）着力聚合改革正能量，实现综合配套改革新突破。改革创新是深圳经济特区的根和魂，不改革就没有出路。我们要以更大的勇气和智慧，敢闯敢试、敢为人先，全面推进综合配套改革，增创科学发展体制机制新优势。一是继续深化经济体制改革。坚持市场化改革取向，进一步理顺政府和市场的关系。深化财税、投融资体制改革，引入市场竞争机制，创新财政支持产业发展方式，增强财政资金的引导和放大作用。加快服务业综合改革试点。打破垄断，降低门槛，简化程序，提高效率，推进社会投资建设项目审批制度改革。加大国有企业改革重组力度，增强国有经济活力。创造公平竞争市场环境，支持非公经济健康发展。继续深化土地管理制度和商事登记制度改革。创新统计方法，探索建立反映经济中心城市辐射带动作用的指标体系，更好地发挥统计在科学发展中的积极作用。积极稳妥推进社区股份公司改革，支持社区经济加快转型升级。二是加快推进社会领域改革。进一步理顺政府和社会的关系，积极发挥行业协会等社会组织作用，逐步形成政社分开、权责明确、依法自治的现代社会组织体制。完善政府主导、可持续的基本公共服务体系。积极探索收入分配制度改革，多渠道增加居民收入。深化户籍制度改革，完善积分入户制度。加快医疗卫生体制改革，创新公立医院管理体制。推进公交等公用事业改革，创新公共服务供给模式，鼓励和引导社会资本参与教育、文化、医疗、养老等事业发展。三是加快行政管理体制改革。完善大部门制改革，建立更加明晰的部门职责体系。深入推进行政审批制度改革。深化公务员分类管理和聘任制改革，规范和完善公务员交流机制。稳妥推进基层管理体制改革。深化事业单位改革，继续开展法定机构试点和机关事业单位雇员制度改革。

　　（四）着力提升开放型经济水平，加快建设国际化城市。开放是提升深圳国际竞争力的必由之路。要实行更加积极主动的开放战略，提高外溢型经济发展水平和质量，强化全国经济中心城市功能。一是加快高端资源集聚。落实支持总部经济发展政策，加大投资推广力度，吸引更多跨国公司和机构在深设立地区总部，引进国内外大型总部企业10家以上。推动引资、引技和引智有机结合，重点引进战略性新兴产业、先进制造业和现代服务业项目。支持企业“走出去”，鼓励优势企业开展海外并购和设立分支机构、研发中心，稳定和扩大海外市场份额。二是全面推进前海大开发大开放。依托香港、服务内地、面向世界，构建引领国际经济合作与竞争的开放高地。用好前海比经济特区更加特殊的先行先试政策，在基础建设、招商引资、产业发展上实现大提速，推进大开发。加快前海商事法制建设，营造法治化国际化营商环境。推动产业准入、优惠目录尽快出台。积极引进现代服务业项目和各类要素交易平台。拓宽境外人民币资金回流渠道，推动跨境人民币业务创新。立足高端，引进30家以上世界500强企业，实现利用外资70亿美元以上。抓紧实施前海综合规划，精心打造重点开发单元。三是提高城市国际化水平。扩大国际交流合作，拓展友城网络，借助侨界、使领馆和深圳国际形象大使等资源，开拓国际交往新渠道。争取在深举办更多的国际高水平赛事和各类展会。深入开展市民讲外语活动，继续推进城市双语标识工作，提高公共媒体外语应用水平。四是优化外贸结构。扩大自主知识产权、自主品牌和高新技术产品出口，拓展海外销售网络和渠道，巩固欧美、香港等重点市场，扩大与东南亚的贸易合作，以更大力度开拓南美、中东、俄罗斯等新兴市场。加快保税区转型升级。大力发展服务贸易，服务贸易占外贸进出口比重达到15%以上。扩大先进技术、关键设备和零部件进口，引进培养具有全球供应链运营能力的企业，努力打造全国重要的进口商品分销中心。积极应对贸易摩擦，支持企业运用WTO规则维护权益。五是加强区域合作。推动深莞惠一体化，加快深汕特别合作区和产业转移园区建设。提升城市交通枢纽功能，启用深圳机场T3航站楼，建成沿江高速、博深高速、厦深铁路，加快穗莞深城际线深圳段、深中通道等重大项目规划建设。实现深圳通与岭南通互联互通。深化深港、深澳合作，加强与台湾地区的交流联系，促进经贸、教育、科技、金融、旅游等领域的互利共赢。开工建设莲塘—香园围口岸和深圳河治理四期工程，加快深港西部快速轨道前期工作。以“一城一园”为重点，扎实推进对口援疆工作。认真完成对口支援西藏、帮扶贵州和省内相关地区等任务。

　　（五）着力推进生态文明建设，打造美丽深圳。环境是生产力，是竞争力，是未来发展的基础，以污染环境和过度消耗自然资源为代价的增长没有前途。要把生态文明建设摆在更加突出的位置，推动深圳实现更长远、更持续、更高质量的发展。市本级在生态环保领域投入223.3亿元，实施一批生态文明建设项目，为建设美丽深圳提供坚实保障。一是优化城市空间开发格局。严格实施城市总体规划和土地利用总体规划，深化陆海统筹，拓展发展空间。制定实施特区一体化新三年计划，今年市本级投资73.6%安排在原特区外。加快光明、坪山、龙华、大鹏新区发展。推动大空港地区、深圳湾—后海总部基地、笋岗—清水河现代服务业基地，以及深圳北站商务区、光明门户区、盐田中心区、坪山中心区等规划建设。推进华强北商圈改造升级。加强土地资源集约节约利用，加大违法建筑查处力度，积极稳妥处理农村城市化历史遗留问题。新开工一批城市更新项目，建立土地整备与新供用地指标挂钩机制，全年整备入库土地18平方公里。二是促进绿色低碳发展和资源节约。加快国际低碳城启动区建设。新开工建设项目率先全面推行绿色建筑标准，继续实施太阳能屋顶计划、LED路灯改造计划和公共建筑合同能源管理。新推广使用新能源汽车1700辆以上。启动编制政府绿色采购目录。积极建设节水型城市，加快推进海水和再生水利用。三是完善城市现代交通体系。增强城市交通枢纽地位，提升全国经济中心城市辐射力。大力构建公交、地铁、轻轨、自行车等低碳交通网络，推进坂银通道、坪盐通道等前期工作，加快轨道三期、现代有轨电车试验线、彩田路北延段等项目建设，鹤洲立交、平安立交建成通车，打通33条断头路，畅通片区微循环和特区一体化交通。完善人行道、自行车道建设，构建步行、自行车与公交便捷接驳系统，推广公共自行车租赁服务，全年新投放公共自行车1万辆以上。加强公交都市建设，发布公交服务指数，提高轨道交通运行效率，公交分担率达到55%。大力发展智能交通，实时发布交通运行指数，着力缓解道路拥堵。四是加强环境综合治理。完善河流整治责任体系，全面推行河长制，实施主要河流生态修复工程，继续改善前海湾、深圳河、龙岗河水质，加快观澜河、茅洲河、坪山河、葵涌河等重点流域综合治理。强化大气污染物跨区域协同控制，完成电厂机组低氮或脱硝改造，强化PM2.5防治工作，实时公布监测数据。加快完善环境保护基础设施，推进污水、污泥、垃圾处理等工程建设，新建200公里以上污水管网。推进建筑垃圾、餐厨垃圾综合利用和生活垃圾减量分类管理。完善市容综合考核机制。继续实施绿化提升行动，加快建设田头山、铁岗—石岩等自然保护区，建成121公里、3.3万亩生态景观林带，新建改造50个公园，开放大鹏半岛国家地质公园。五是加强生态文明制度建设。完善基本生态控制线管理。健全环境经济政策体系，推行绿色信贷和环境污染责任保险制度。全面开展清洁生产审核，探索建立低碳产品的标识和认证制度，从严控制产业能耗、环保准入门槛。开展碳排放权、排污权交易，完善环境资源有偿使用的市场机制。加强环保实绩考核。实施最严格措施，加强水资源管理。通过全市人民的共同努力，使深圳的天更蓝、地更绿、水更净、空气更清新！

　　（六）着力增进民生福祉，构建和谐幸福家园。保障和改善民生是政府一切工作的根本导向。要把人民生活质量提升、幸福感不断增强作为经济发展的最终目的，多谋民生之利，多解民生之忧，让市民更幸福、城市更包容、社会更温暖。一是加大民生投入。坚持尽力而为、量力而行，保基本、可持续，今年市财政安排重点民生领域支出增长13.5%，其中教育、医疗卫生、公共交通分别增长20.5%、32.7%和27.9%。投资18亿元启动优质饮用水入户工程。新建天然气市政管道105公里，增加10万户管道天然气居民用户。二是提供更好的教育服务。加大教育投入，实行公办中小学运行经费生均拨款制度。加快提供优质学位，新改扩建中小学校18所，增加公办学位1.85万个。新增幼儿园学位2万个，规范学前教育管理，在有条件的社区至少建有一所普惠性幼儿园。实施学生综合素养提升工程。加快发展高等教育，支持北大、清华、哈工大深圳研究生院和深圳大学提升教学科研水平，加快推进南方科技大学、香港中文大学（深圳）发展，高起点建设2—3所专业化、国际化特色学院。大力培养高技能人才，支持深圳职业技术学院、信息职业技术学院等改革发展。全面开展职工技能培训，提高职业技能资格证书覆盖率。实施全民素质提升计划。三是让市民得到更可靠的社会保障。进一步完善多层次的社会保障体系。实施积极的就业政策，多渠道促进就业。构建和谐劳动关系。落实国家房地产调控政策，加大住房保障力度，全年提供保障性住房2.5万套。健全居民最低生活保障标准调整机制，着力提高困难群体、特殊群体和优抚群体的生活保障水平。加快推进市儿童福利院建设。积极发展残疾人事业。大力发展老龄服务事业和产业，加快推进市老人综合服务中心建设，出台养老设施专项规划。支持发展慈善事业，办好第二届慈展会。四是提供更好的医疗卫生服务。建成市儿童医院住院综合大楼、市第二人民医院综合大楼，推进新明医院、聚龙医院等重点项目建设。支持民营医疗机构发展，推广网络健康服务，促进优质卫生资源均衡布局和共享。积极引进名医和重点专科团队，深入推进社康中心标准化建设，壮大全科医生队伍，大力推广家庭医生服务，加强市民健康管理和医疗保健服务。加强医德医风建设。完善医疗急救、卫生应急管理体系。提升计生服务质量。五是实施科技惠民工程。加强现代科技在医疗卫生、社会保障、教育文化、交通出行、公共安全、食品药品等领域的应用。完善城市信息基础设施，提高互联网、家庭宽带普及率和无线宽带覆盖率，光纤入户增加到100万户。加强网络信息保护，维护信息安全。六是进一步提高社会建设水平。推进全国社会管理综合试点。继续实施“织网工程”和“风景林工程”，整合基层管理和服务资源，为市民提供更优质的社区服务。完善信访制度，健全矛盾纠纷调解工作体系，畅通和规范群众诉求表达、利益协调、权益保障渠道。深入开展“三打两建”工作，着力完善社会信用体系和市场监管体系。加大社会治安综合治理力度，增强防范和打击犯罪能力，促进社会治安持续好转，打造平安深圳。强化公共安全体系建设，正式发布深圳公共安全白皮书，加强安全生产和消防安全，持续开展泥头车等重点领域专项整治。加快推进气象现代化建设，完善减灾防灾和综合应急救援体系。强化食品安全管理，加快建立食品安全追溯系统和黑名单制度，坚决打击制假售假，提高检测能力，加大抽检力度，严管重罚，加强对食品生产、加工、流通、消费全过程监管。推进药品电子监管，提升药品安全保障能力。规范发展社会组织，建立政府行政监管、社会公众监督和社会组织自律相结合的综合监管体系，支持工会、共青团、妇联等群团组织发展，加强双拥共建工作和国防动员体系建设。

　　（七）着力建设文化强市，增强城市软实力。文化是凝聚力和创造力的源泉，是城市内涵和品位的重要体现。要大力加强文化建设，培育城市人文精神，提升城市整体实力。一是丰富市民文化生活。大力发展文化事业，新开工深圳书城宝安城、坪山新区文化综合体和国际网球中心、观澜版画艺术博物馆等一批文化项目，加快当代艺术馆和城市规划馆、大鹏所城二期保护等重大项目建设。完善基层公共文体设施，继续推动公共文体场馆免费或优惠开放，积极创建国家公共文化服务体系示范区。实施文化惠民工程，深入开展公益文化活动进校园、进社区、进企业，继续办好深圳读书月、创意十二月、市民文化大讲堂、公园文化节、万场电影惠百姓等文化活动。大力发展哲学社会科学，鼓励学术性文化活动。推进文化菁英集聚工程，推出更多原创文化精品。深入开展全民健身活动。鼓励发展竞技体育，积极打造有影响力的竞技队伍。二是提升文化产业核心竞争力。积极引进文化产业重大项目，加快国家动漫产业基地建设，推进华强文化创意基地、华谊兄弟文化城、前海文化创意产业基地、福田新媒体产业园等项目前期工作。推动文化与科技、金融、时尚、旅游等深度融合，发展新型文化业态。壮大创意人才队伍，积极发展文化创意产业，打造一批具有核心竞争力的文化龙头企业。发挥文博会等国家级文化产业发展平台的作用，进一步增强文化产业集聚辐射功能，促进更多文化产品走向世界，争当文化产业发展领头羊。三是塑造深圳时代风尚。深入开展社会主义核心价值观教育，大力弘扬新时期“广东精神”、“大运精神”和深圳“十大观念”。营造劳动光荣、创造伟大的社会氛围，倡导以阳光心态面对人生、以辛勤劳动创造生活、以感恩情怀融入社会。注重人文关怀和心理疏导，加强社会公德、职业道德、家庭美德、个人品德教育，全面实施深圳经济特区文明行为促进条例。加强政务诚信、商务诚信、社会诚信建设。推进志愿者之城、爱心之城、慈善之城建设。推广“社工＋义工”服务模式，扩大公民在社会管理和公共服务中的参与度，增强公民社会责任感和城市凝聚力。

　　（八）着力加强自身建设，努力建设廉洁高效的法治政府。做好今年各项工作，必须以改革创新精神加强政府自身建设，坚持从严治政，突出“干”字当头，着力提高服务能力。一是坚持依法行政。认真接受市人大及其常委会的法律监督、工作监督和市政协的民主监督，严格执行市人大及其常委会决议决定，切实做好建议提案办理工作。主动听取各民主党派、工商联、各人民团体和各界人士的意见建议。严格依法行政，努力建设法治政府。加强法制宣传教育，增强全社会学法、遵法、守法、用法意识，维护法律权威，建设法治城市。二是大力改进工作作风。切实落实中央改进工作作风的八项规定和省委、市委的要求，开展“弘扬改革创新精神、树立优良工作作风”学习教育活动，力戒形式主义、官僚主义，干实事，敢作为，勇担当。深入实际、深入基层，认真办好事关群众切身利益的民生实事。加强明查暗访，坚决纠治作风不实、庸懒散奢的不良风气。三是持续提高行政效能。加强电子政务建设，强化信息资源共享，严格行政电子监察。进一步优化政务流程，精简审批事项。完善绩效考核指标体系，加强责任追究，提高政府效能。四是切实抓好廉政建设。全面推进源头治理、制度防腐。强化财政资金监管，坚决反对铺张浪费。加强审计与监察，严厉查处违法违纪案件，保持惩治腐败高压态势，营造风清气正的发展环境，建设廉洁城市。

　　各位代表，深圳正以更加坚定的步伐、更加昂扬的斗志走在科学发展的道路上。让我们高举中国特色社会主义伟大旗帜，认真贯彻落实党的十八大和习近平总书记视察广东深圳重要讲话精神，在党中央国务院、省委省政府和市委的坚强领导下，解放思想，振奋精神，凝聚力量，扎实工作，用我们的勤劳和智慧，共同创造深圳经济特区更加美好的未来！
